# Evaluating the Acceptability of Virtual Preventive Genetic Counseling Supporting Adult Primary Care Practices

**DOI:** 10.1007/s11606-025-10087-7

**Published:** 2026-01-05

**Authors:** Leland E. Hull, Suzanne Brodney, Susan Regan, Olivia Benson, Katherine L. Gallagher, Caylin Marotta, Olivia Ritchie, Kristen M. Shannon, Anna Verwillow, Heidi L. Rehm, Jennifer S. Haas

**Affiliations:** 1 Department of Medicine, Massachusetts General Hospital, Boston, MA USA; 2 Havard Medical School, Boston, MA USA; 3 The Broad Institute of MIT and Havard, Cambridge, MA USA; 4 The MGH Institute of Health Professions, Boston, MA USA

**Keywords:** genetic counseling, genetic testing, primary care

## Abstract

**Background:**

Scaling access to preventive genetics services requires implementation of new service delivery models. We launched and evaluated a new virtual clinical service, the Preventive Genetic Counseling Service (PGCS), to support the genetic counseling needs of a network of adult primary care practices.

**Objective:**

To determine the acceptability of a new virtual genetic counseling service to referring primary care clinicians and patients.

**Design:**

A mixed-methods evaluation of the first year of clinical service operations.

**Subjects:**

Referring primary care providers to and patients seen by the preventive genetic counseling service from July 2023 through June 2024.

**Approach:**

Mixed-methods analyses were used to synthesize quantitative findings from a clinical patient tracking system and patient surveys, as well as rapid qualitative analysis of multi-referring clinician interviews.

**Key Results:**

Of 281 patients referred in the first year, 251 were seen. The most common visit reasons were for genetic counseling regarding predisposition to breast and related cancers (203/251, 82%), preconception counseling (31/251, 12%), and lastly for interpretation of direct-to-consumer genetic testing or other (17/251, 7%). Most patients seen completed genetic testing (152/251). Most patients returned to the care of their primary care clinician without the need for specialty care follow-up after their visit (187/251, 74%). Patients who completed an after-visit survey (*n* = 73 of 202 invited, response rate 36%) reported high satisfaction with the service, as indicated by a net promoter score of 80. Interviews were completed with 10 clinicians who referred multiple patients. They emphasized their satisfaction with the clinical service, the importance of supporting primary care clinicians in genetics, and the value of communication and clear handoffs between this service and primary care.

**Conclusions:**

In its first year of operations, the PGCS model was acceptable to referring clinicians and patients.

**Supplementary Information:**

The online version contains supplementary material available at 10.1007/s11606-025-10087-7.

## BACKGROUND

Alternative service delivery models are needed to facilitate access to evidence-based preventive genetics care in a primary care setting. The indications for preventive genetic testing continue to expand such that patient demand will increasingly outpace the supply of genetics subspecialists.^1^ For example, in 2019 the U.S. Preventive Services Task Force increased the population who should consider genetic counseling and/or testing for a genetic predisposition to the hereditary breast and ovarian cancer syndrome.^2^ Additionally, evidence is growing to support broader implementation of preconception reproductive genetic carrier screening (RGCS),^3,4^ as opposed to performing carrier screening once a patient is already pregnant. The American College of Medical Genetics (ACMG) has recommended increasing the number of conditions to routinely be included in carrier screening for recessive conditions that could be passed on to future children. Finally, modeling studies suggest that population-based screening of young adults for a core set of monogenic conditions (CDC Tier 1 conditions) would be cost-effective^5^ and clinical biobanks have uncovered actionable genetics for large numbers of patients,^6^ setting the stage for consideration of population-based genetic screening programs.

Given that primary care clinicians (PCCs) serve as the first point of contact for patients seeking preventive services, including cancer screening and reproductive counseling,^7^ it has been suggested that PCCs will need to incorporate preventive genetics care into their practice. However, this simply has not been successful. The U.S. faces a growing primary care clinician shortage,^8^ further straining PCC bandwidth. There have also historically been several barriers to engaging primary care clinicians in genetics care, including healthcare system and access constraints and insufficient knowledge and comfort with genetics.^9^

While PCCs cannot be expected to navigate genetics care unsupported, thoughtful and efficient use of genetics specialists is necessary to preserve access given genetic services are limited and unevenly distributed.^10,11^ Integrating genetic counseling in primary care is one way to address this conundrum.^12^ We introduce a novel preventive genetic counseling service providing virtual genetic counselor support to primary care clinicians for a limited “menu” of high-demand indications. The service launched in 2023, with the goals of tackling current health system access challenges, engaging genetic counselors as part of the extended primary care team, and preparing for anticipated increased future preventive genetics demand. We conducted a mixed-methods evaluation of a novel preventive genetic counseling service pilot to evaluate the clinical impact and acceptability of the service to patients and referring clinicians during its first year.

## METHODS

This study was deemed exempt by the Mass General Brigham Institutional Review Board (Protocol # 2023P000751). A waiver of written informed consent was granted. Survey participants indicated consent at the start of their survey, and referring providers provided verbal consent at the start of their interview.

### The Preventive Genetic Counseling Service Intervention

Prior to the launch of the Preventive Genetic Counseling Service (PGCS), patients seeking any genetic counseling were routinely referred to disparate subspecialty clinics based on their specific concerns. Access was limited and wait times were long (months to even a year or more) depending on the subspecialists’ availability. The PGCS, which launched in July 2023, instead saw patients with a system-affiliated PCC for a set menu of high demand but limited access indications: (1) genetic counseling of unaffected patients for predisposition to breast and related cancers (e.g., pancreatic, ovarian, prostate), (2) coordination of preconception carrier screening for both individuals and reproductive couples, and (3) first-pass assistance with interpretation of direct-to-consumer genetic test results when patients presented to their PCC with these results. The rationale behind this menu was that increasingly large populations of patients met clinical guidelines for genetic counseling and/or testing in these scenarios; access to genetic counseling for these patients was limited, and diverting patients from subspecialty clinics for these indications could improve access to subspecialists for more complicated diagnostic cases and positive test result management.

**Figure 1:**
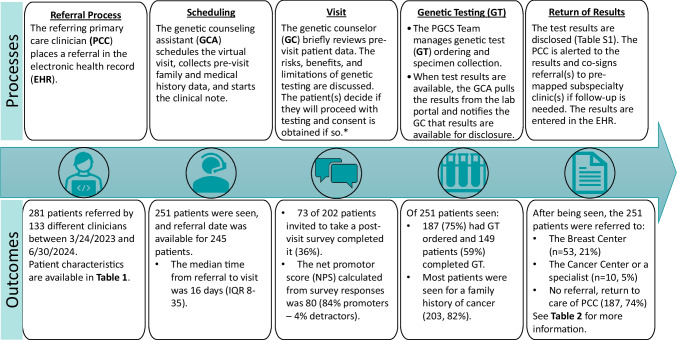
Clinical workflow and first-year process outcomes. *Genetic counseling visits were billed using CPT code 96040 during the study period. This billing code covers each 30 minutes of face-to-face genetic counseling, with a minimum of 16 minutes of face-to-face genetic counseling provided to bill. Of note, as of January 1, 2025, 96040 has been replaced with 96041, which allows billing for face-to-face and non-face-to-face time spent by the genetic counselor.

The PGCS consisted of the following team members: (1) a dedicated genetic counselor (GC) (0.25 FTE dedicated to the service, A.V.), (2) a genetic counseling assistant (GCA), who assists in scheduling patients, facilitating genetic test ordering, specimen collection, tracking of genetic tests being processed, and disclosing some test results (0.2 FTE dedicated to service, O.B. and later O.R.), and (3) a physician director overseeing patient visits (L.H.). The general workflow of the PGCS is pictured in Fig. 1, and specific workflows for patients seen by referral indication are detailed in the Supplement (Figure S1). Patients were either referred to the service by their PCC or identified proactively by the GCA by reviewing subspecialty clinic queues and redirecting appropriate patients to the PGCS. The GCA contacted patients scheduled for a visit to provide information about their family history, personal medical history, and history of personal or familial genetic testing in advance of the visit via electronic questionnaire or phone, and a note was started in the electronic health record incorporating this information for the genetic counselor to review. Genetic counseling visits were therefore meant to focus on brief corroboration and/or clarification of pre-visit data collection and then focused counseling about the risks/benefits/limitations and patient preferences regarding genetic testing for disease risk. Compared to traditional hour-long genetic counseling visits, the PGCS genetic counselor was scheduled to see new patients for 30 minutes, so she could see up to 7 patients per half-day clinical session. Patients’ insurance was billed for their visit using CPT Code 96040, which is a time-based code for use by trained genetic counselors without requiring the presence of a physician.^13^ It can be used to bill for 30 minutes of face-to-face genetic counseling time with a patient or family.

Patients seeking cancer predisposition testing were offered several options for multi-gene cancer panels with up to 85 genes and decided on a panel using shared decision making with the genetic counselor. Patients seeking reproductive genetic carrier screening were offered the academic medical center’s standard expanded carrier screening panel of up to 267 conditions. Additional testing could be added based on the genetic counselor’s assessment, if necessary, and all testing ordered was coordinated to coincide with the offerings of specialty clinics across the health system for standardization.

Patients electing to proceed with genetic testing completed an electronic consent form. All testing was coordinated remotely using mailed saliva kits. Patient results were disclosed after results returned (usually 3–4 weeks) by the GC if positive and/or if a more nuanced discussion of the results was needed. Results were returned by the GCA if testing was negative. The PGCS team consulted with subspecialist working group colleagues to create specific mappings of how to direct patients after their PGCS visit based on genetic testing decisions, results, and disease risk factors. Patients with actionable test results would be referred to the appropriate subspecialty clinic, such as the system’s cancer center, medical genetics group, or reproductive subspecialists, for further management. Patients with a ≥ 20% lifetime risk of breast cancer calculated using the Tyrer-Cuzick score^14^ or other high-risk feature (relative with early diagnosis, history of chest radiation) were offered a referral to the health system’s breast center for consideration of early screening and/or enhanced screening protocols (e.g., consideration of breast MRI), even if genetic testing was negative or declined.^15^ Patients deemed not to need specialty physician evaluation returned to their primary care physician for continued preventive care. Novel aspects of this service relative to the prior institutional model of traditional referral to subspecialty services are listed in Box 1. 

 Box 1: Novel aspects of the PGCS delivery model compared to the existing subspecialist referral model

**Table:** 

• Created a “menu” of reasons for referral to streamline clinic processes (decrease heterogeneity) that could be updated based on changing genetics needs over time
• Used pre-visit data collection to increase the efficiency of GC appointments
• Engaged primary care clinicians as partners in the genetic counseling process via co-signing of teed up recommendations and/or referrals based on triage and GC assessment with minimal lift
• Passively educated referring PCCs about the genetic counseling process through being copied on chart notes and results after disclosure so they may follow their patient’s genetic counseling journey
• Preserved subspecialists for complex cases, since only high risk patients or patients with a positive genetic test result were referred on to a subspecialist (top-of-license care)
• Increased GC billable visits due to system efficiency so that GC could see two patients in an hour (previously 1 patient/hour)
• Decreased use of multiple systems specialists since patients could request genetic testing for more than one reason at a single visit (e.g., a young woman seen at the PGCS could pursue cancer predisposition testing and preconception carrier screening during the same encounter, as opposed to seeing both cancer and obstetrical genetics groups)
• Engaged genetic counseling assistants to facilitate pre-visit data collection and return negative test results, preserving GC time for top-of-license care

### Study Population and Data Sources

The study population included the patients referred to the PGCS between 03/24/2023 and 6/30/2024 and their referring providers. Three primary data sources were analyzed: (1) a clinical tracker of patients referred to the clinic, (2) a survey administered to patients after their visit, and (3) qualitative interviews with multi-referring clinicians. We describe data acquisition and analyses for each of the three sources below.

### Clinical Tracking and Patient Survey Data

Study data were collected and managed using REDCap electronic data capture tools hosted at Massachusetts General Hospital (MGH).^16,17^ REDCap (Research Electronic Data Capture) is a secure, web-based software platform designed to support data capture for research studies. A clinical tracker established in REDCap was set up at the launch of the clinical service to monitor referrals, clinic visits, tracking of genetic testing and results disclosures, and follow-up recommendations. The clinical tracker was maintained longitudinally by a genetic counseling assistant (O.B. and O.R.). The fields collected in the clinical tracker are available in the Supplement.

Second, patients who completed a visit with the PGCS were sent a survey through REDCap based on the outcome of their visit. The participants were divided into three groups based on the outcome of their visit. First, patients who elected to pursue genetic testing and completed testing within 3 months were sent a survey after their genetic test results were disclosed. Patients who did not have genetic testing ordered after their visit were sent a survey immediately after their visit. Finally, patients who stated they would like to pursue genetic testing, but who had not completed testing within 3 months of their visit, were sent a survey at the 3-month mark. All patients were asked about their motivation for genetic counseling,^18^ their experiences with the service,^19,20^ and about their demographics.^19,21,22^ Additional questions were tailored based on whether the patient completed testing.^23,24^ All survey versions can be found in the Supplement.

REDCap data used in the clinical tracker and survey data analyses were downloaded on 11/13/2024. Descriptive analyses were conducted using Stata Statistical Software, version 18 (StataCorp, 2023) STATA.

### Interviews of Multi-referring Clinicians

To probe clinicians more deeply about their experiences with the service, focusing on understanding the model’s acceptability and areas of future need, semi-structured virtual video interviews were conducted with referring primary care clinicians. To elicit richer responses, clinicians who had referred at least two patients to the clinic by 3/18/24 were eligible to participate. The interview team developed an interview guide, which is available in the Supplement. Questions were designed by the team and piloted internally to address specific constructs related to the perceived preventive genetics needs of the patient and clinician population, experience with the PGCS, acceptability of the PGCS,^25^ perceptions of genetic counseling and genetic testing following the use of the service, barriers, alternatives and desired improvements to the service, and open-ended comments. The interview guide was tested by the members of the clinical team and refined iteratively. Clinicians meeting inclusion criteria were identified in the REDCap clinical tracker and recruited via email. Clinicians were offered a $50 Amazon gift card as remuneration.

Interviews were conducted in April and May of 2024 until thematic saturation was reached, as indicated by an absence of new insights and repetitive responses to our questions.^26^ Participants were scheduled for a 30-minute semi-structured virtual video interview conducted using Microsoft Teams, which allows for video interview recording and provides live transcription. Verbal consent to participate was obtained at the start of the interview. S.B., a health services researcher, conducted the interviews. After each interview, the transcripts and videos were saved to allow for cleaning and revisiting the transcripts in case of discrepancies.

The interview data was analyzed using rapid qualitative analysis methods. Specifically, a matrix approach was used.^27–29^ Given that our interview guide questions were designed to address pre-specified constructs of interest with each question, the interview transcripts were broken up into a matrix where each participant’s responses were organized by question. L.H. and S.B. each individually read through and inductively coded all the participants’ responses to each question; they would then meet weekly to reconcile their codes. After all individual questions were coded, emergent themes spanning the reconciled codes were identified individually and then reconciled together.

## RESULTS

### Service Delivery Metrics

In the first year of operations, 281 patients were referred to the PGCS and 251 completed a visit (Fig. 1). The mean age of patients referred was 43 years, and most referrals were for female (89%) patients who self-reported White race (80%) (Table 1). Patients referred to the service were younger and disproportionately female compared to the referring practices, which is not surprising given the indications for referral (Table S1). A family history of cancer was the predominant reason for referrals (81%), although 12% of referrals were for preconception counseling with carrier screening and 6% for interpretation of direct-to-consumer genetic testing (Table 1).

**Table 1:** Characteristics of Patients Referred to the PGCS (*N* = 281)

	Visit completed *n* = 251	Visit not completed *n* = 30	All referrals *N* = 281
Mean age (SD)	43.1 (15.1)	42.4 (20.6)	43.0 (15.7)
Female *N (%)*	223 (89)	26 (87)	249 (89)
Race			
Asian* N (%)*	13 (5)	Suppressed*	Suppressed*
Black* N (%)*	17 (7)	Suppressed*	Suppressed*
White* N (%)*	202 (80)	23 (77)	225 (80)
Other/unknown race* N (%)*	20 (8)	Suppressed*	Suppressed*
Hispanic ethnicity (any race)* N (%)*	21 (8)	Suppressed*	Suppressed*
Indication for referral			
Family history of breast (or other) cancer *N (%)*	203 (82)	26 (87)	228 (81)
Preconception carrier screening *N (%)*	31 (12)	Suppressed*	Suppressed*
Interpretation of DTC testing *N (%)*	17 (7)	Suppressed*	Suppressed*

*Cells with counts 5 have been suppressed to protect participant confidentiality. Percentages for suppressed values are not shown. Column totals may not equal the sum of visible values due to suppression

Most patients seen had genetic testing ordered (187 of 251), and most completed testing (*n* = 152) (Table 2). Although the majority of tests were negative, reporting no pathogenic variants, likely pathogenic variants, or variants of uncertain significance (107 of 171 tests ordered), 15 pathogenic variants were identified in cancer predisposition genes and 12 pathogenic variants were identified on carrier screens (Table 2).

**Table 2:** Genetic Testing Orders and Results for Patients Seen in the PGCS

	Cancer test orders	Carrier screening test orders	Other genetic tests	All
Genetic tests ordered* N (%)* *Patients*	171 (83) *168*	30 (14) *30*	6 (3) *5*	207 *187*
Genetic tests completed* N (%)* *Patients*	138 (81) *135*	27 (16) *27*	6 (4) *5*	171 *152*
# Pathogenic/likely pathogenic variants N (%)*	15 (11)	12 (44)**	3 (50)	30
# Variants of uncertain significance (VUS)	38 (28)	***	***	38
# Tests with no P/LP/VUS results (fully negative)	89 (64)	15 (56)	3 (50)	107

*P/LP* pathogenic/likely pathogenic, *VUS* variant of uncertain significance. ^*^Patients may have more than 1 test result. ^**^Although 12 pathogenic variants were identified, no at-risk couples (couples who both carry a pathogenic variant implicated in the same autosomal recessive disease) were identified. ^***^Variants of uncertain significance are not routinely reported for patients undergoing carrier screening, direct-to-consumer genetic testing, or proactive health screens

**Figure 2:**
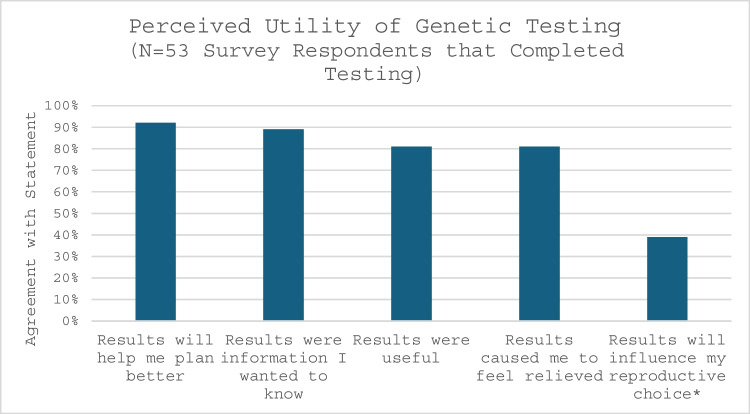
Patient survey results—satisfaction with service delivery and perceived utility of genetic testing. *Analysis limited to respondents age < 40 years.

Surveys were sent to patients who completed a visit (*N* = 202) to assess their satisfaction with the service, and additional questions were tailored based on whether they completed genetic testing or not (Fig. 1). Surveys were completed by 73 patients (36% response rate). Respondents indicated patient satisfaction with the service (Figure S2) and favorable experience ratings (Net Promoter Score^30^ of 80, Fig. 1). Survey respondents who completed genetic testing (*N* = 57) endorsed its utility in multiple domains (Fig. 2).

### Qualitative Interviews

Of 35 multi-referring clinicians who were sent at least one recruitment email, 10 participated (29%) in an interview. Interviewed clinicians included 8 females and 2 males from 7 different hospital-affiliated primary care practices who were predominantly MDs (*n* = 9), although one nurse practitioner participated. Interviewed clinicians were generally positive about the service, although areas for improvement and growth were also noted (Table S2). Three themes emerged from the clinician interviews: (1) general satisfaction with the PGCS as a clinical service, (2) the need to support primary care clinicians, and (3) the value of communication.

With regard to the first theme (satisfaction), providers indicated their general satisfaction with the service. Some indicated that while genetics evaluations were not always common or high volume in their practice, having the service available provided a sense of support to help address concerns as they arose:

It’s [not] every week thing that I’m sending someone to the genetic counseling service. But when there is that […] person that comes in […] with a question or concern, I would say that it’s super helpful and I think seems to fit a need you know in the […] system to help people answer those questions that I think for most of us just don’t have the knowledge base to answer.—Participant #8

Yeah, I think it’s helpful for my patient, yeah. And then obviously my patient’s happy, I’m feeling like […] I’m doing a good job. So yeah, it’s [a] benefit.—Participant # 6

I would love for this [service] to continue. It’s easy access to get into. It’s good care and it helps people transition to what they need or if they don’t need increased screening.—Participant #1

With regard to the theme of supporting primary care clinicians, clinicians expressed that even if they had the desire to provide preventive genetics care, they would not feel equipped to do so, citing several concerns including gaps in perceived knowledge, a rapidly evolving genetics field, and insufficient time and support to provide preventive genetics care themselves.

I just think primary care is too busy. Yeah. ...We’re very grateful honestly, to have this service. It’s complex…genetic testing keeps changing as new genes are discovered. And I just think it would be very hard to add this on to what we do in primary care.—Participant # 3

And an important part that we didn’t speak about is when the test does come back, they have someone in the Genetic Counseling Service go through the tests… I think that also is very helpful for me that the patient has that discussion with someone who has specialty expertise.—Participant #5

Although PCCs shared that they would not be interested in providing genetics care themselves, they expressed satisfaction with the support the PGCS provided them to care for patients seen by the service.

You know, I’m glad that we have a way of pursuing these questions because I think in the past, we didn’t always know what to do. So, I think it’s nice that we’ve got an option for patients to actually explore genetic risk factors and […] apply the right screening strategy and make sure that that we’re catching cancers early. So, I do like having that option.—Participant #2

It feels very like a well-supported service – it supports us as PCP as well. Knowing that a lot of us don’t have a lot of in-depth knowledge of genetic things like this and how to manage them. It’s reassuring to know that I’m not just sending somebody for testing and getting a report back and then having to figure out what to do with it.—Participant 7

Finally, providers emphasized the value of closed-loop communication and clear handoffs when referring patients.

The most important thing [to me] is that if I place a referral and somebody’s evaluated and there’s something to do that that it comes back to me. I just want to know. I just want to recognize that that it’s been put back on my plate.—Participant 7

The clinicians’ noted potential future directions both for expanding clinical offerings and improving operational processes. These included increased resources for specific diseases (Alzheimer’s disease, hemochromatosis), increased education, focusing on equitable access to genetics services, expanded offerings, and further refining communication and referral practices (Table S2).

With my large patient panel, [referring to the PGCS]may not be something that I think of right away. And maybe if there was a way to kind of notify us of patients who we might consider that might be helpful?—Participant #10

Equity is always on my mind. So I guess there’s always the question about are we and doing a good job of making sure that this is an equitable process. So we’re making sure that you know everyone you know.—Participant #3

## DISCUSSION

In this article, we describe the launch and first year experience of a novel care delivery model – a virtual preventive genetic counseling service paired with PCCs, meant to provide efficient and accessible genetic counseling for a set of indications more commonly encountered in adult primary care. Although other models for integrating genetics in primary care have been described elsewhere,^31^ our model uniquely used a virtual-only approach to allow a genetic counselor to support multiple clinics across the health system. Limiting patient referrals to a narrow menu of preventive concerns allowed our genetic counselor to see greater numbers of patients, offload subspecialist wait lists for more complex or diagnostic cases, and capitalize on the support of a genetic counseling assistant. This allowed all individuals, from primary care clinician to genetic counselor to specialist, to work at the top of their license. Our findings suggest that this model was acceptable and desirable to both referring physicians and patients.

However, despite the acceptability of this model to key stakeholder groups, significant challenges for this model and others integrating genetic counseling in primary care persist. First, reimbursement of genetic counseling services remains a challenge. Genetic counselors are not recognized as healthcare providers by the Centers for Medicare and Medicaid Services, which limits reimbursement opportunities, and attempts to introduce legislation to expand Medicare coverage for genetic counseling services have not moved forward.^32,33^ A review of reimbursement for genetic counseling from 2010 to 2018 found an overall downward trend in reimbursement of GC services, including by commercial payors.^34^ Although the introduction of a new genetic counseling CPT® code, 96041, in 2025 allows genetic counselors to account for additional, non-face-to-face effort on the date of service, and is a step forward for accounting for the total time spent by genetic counselors on patient care,^35^ lack of reimbursement by different payors remains a significant hurdle.

Additionally, this service model depended on primary care clinicians to identify eligible patients and refer them to the service. Given that it has been well-established that clinician biases and relying on clinicians to identify indications for genetic counseling and genetic testing referral can result in inequitable genetic counseling and testing for evidence-based indications,^36^ and that evidence-based preventive genetic services are underutilized,^37,38^ moving towards a more proactive approach to identify patients who would benefit from the service would be desirable. This would also help to address some of the concerns about equitable access raised by the referring clinicians during our interviews. Proactive screening of patients for health concerns at preventive visits^39^ and proactive population outreach to increase the uptake of evidence-based screenings^40^ are well-established concepts in primary care medicine and often facilitated by care teams. Reimagining the role of the genetic counseling assistant as a population health manager and incorporating technology such as Chatbots to proactively identify and recommend genetic counseling referral for patients on a population scale^41^ are exciting near-term opportunities for future service improvement. 

Several limitations of this descriptive study should be noted. First, there was likely some selection bias in recruiting both survey participants and interviewing referring providers. The response rate for the patient survey was low (36%) and could have been skewed towards those with stronger positive or negative experiences. Positive reactions to the clinical model might not be fully attributable to the actual service delivery but might also reflect perceived relative improvements compared to former referral processes and access challenges. Another limitation is that providers were recruited for interviews only if they had made multiple referrals to the service, with the goal of identifying providers with more experience with the service for richer results. However, it is also likely that these providers were sufficiently satisfied with their initial referral experience to refer again. As genetic service delivery in primary care continues to grow and evolve, assessing the acceptability of evolving service delivery will be crucial to refining scalable and sustainable models.

## Supplementary Information

Below is the link to the electronic supplementary material.

ESM1 (DOCX 263 KB)

## Data Availability

The datasets generated and analyzed during the current study are not publicly available because participant consent for data sharing in open-access repositories was not obtained. Deidentified data may be available from the corresponding author on reasonable request and with appropriate Institutional Review Board approval.
