# Supplementary material for: Evaluating the Acceptability of Virtual Preventive Genetic Counseling Supporting Adult Primary Care Practices
Source: J Gen Intern Med. 2026 Jan 5;41(9):2489–97. doi: 10.1007/s11606-025-10087-7 (PMC13304015; doi:10.1007/s11606-025-10087-7)
Supplement: Supplementary file 1 — (DOCX 263 KB) [file 11606_2025_10087_MOESM1_ESM.docx]

**Supplementary Materials**

Contents

[Supplementary Figure 1. Genetic counseling workflows specific to referral indication 2](#_Toc212447190)

[Supplementary Table 1. Comparison of demographics of referring primary care practices and those referred to the Preventive Genetic Counseling Service. 4](#_Toc212447191)

[Supplementary Figure 2. Patient Satisfaction Survey Data 5](#_Toc212447192)

[Supplementary Table 2. High referring provider interview results organized by constructs assessed and cross construct emergent themes. 6](#_Toc212447193)

[PGCS Clinical Tracker Data Collection Fields 9](#_Toc212447194)

[Patient-facing survey 20](#_Toc212447195)

[Qualitative Interview Guide 25](#_Toc212447196)

[COREQ Checklist 29](#_Toc212447197)

Supplementary Figure 1. Genetic counseling workflows specific to referral indication.


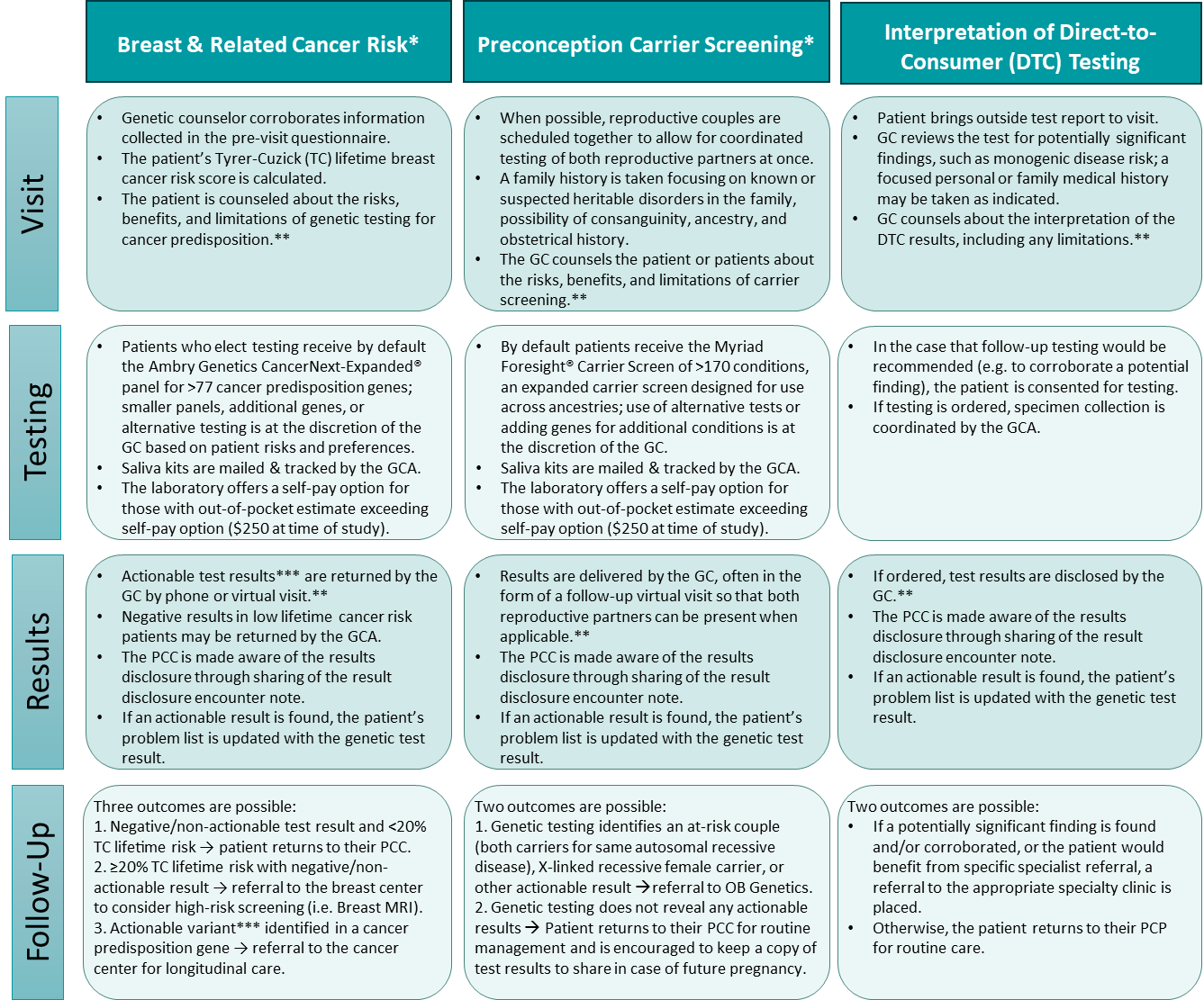


Abbreviations: GC = genetic counselor; GCA = genetic counseling assistant; OB = obstetric; PCC primary care clinician.

*At times patients request both cancer predisposition testing and preconception carrier screening, and the GC tries to accommodate both requests and consent to order multiple tests in this circumstance.

**Genetic Counseling visits were billed using CPT code 96040 during the study period. This billing code covers each 30 minutes of face-to-face genetic counseling, with a minimum of 16 minutes of face-to-face genetic counseling provided to bill. Of note, as of January 1, 2025, 96040 has been replaced with 96041, which allows billing for face-to-face and non-face-to-face time spent by the genetic counselor on day of service. Given this change, especially when the genetic counselor needs to do additional research about a specific test result, virtual visits to return results frequently meet the revised genetic counseling billing code requirements. A supervising physician is required to be billed under for the genetic counselor.

***Genetic variants are reported on a spectrum from pathogenic to benign, depending on available evidence. Depending on the clinical context, variants of uncertain pathogenicity may or may not require additional follow-up. Therefore, actionable variants may include variants with known, likely, or possible pathogenicity depending on the clinical circumstance.

# Supplementary Table 1. Comparison of demographics of referring primary care practices and those referred to the Preventive Genetic Counseling Service.

|  | Demographics of patients from referring primary care practices*  N=40176 (%) | Referrals to the Preventive Genetic Counseling Service  N=281 (%) |
| --- | --- | --- |
| Mean age (SD) | 57.3 (18) | 43.0 (15.7) |
| Female *N (%)* | 21285 (53) | 249 (89) |
| Race |  |  |
| Asian *N (%)* | 3421 (9) | 13 (5) |
| Black *N (%)* | 2798 (7) | 20 (7) |
| White *N (%)* | 31029 (77) | 225 (80) |
| Other/Unknown race *N (%)* | 2928 (7) | 24 (8) |
| Hispanic ethnicity (any race) *N (%)* | 2431 (6) | 24 (9) |

*The three primary care practices from which referrals were accepted before expanding to access system-wide referrals are used for comparison.

# Supplementary Figure 2. Patient Satisfaction Survey Data

**The genetic counseling assistant (GCA) served as the scheduling staff for the clinic.

# Supplementary Table 2. High referring provider interview results organized by constructs assessed and cross construct emergent themes.

| **Constructs Assessed** | **Summary of Interview Findings by Construct Assessed and Relationship to Emergent Themes** |
| --- | --- |
| Theme 1: Satisfaction with the PGCS as clinical service (Acceptability of the Service)  Theme 2: Supporting primary care clinicians  Theme 3: Value of communication and clear handoffs | |
| Intervention Coherence | - There was general agreement that the process followed by the clinic was clear to the patient. (Theme 3) - Some referring clinicians had confusion about 1) when to refer to this genetic counseling service vs. other specialty services (e.g. the Cancer Center) and 2) to what extent does this service provide longitudinal care vs. a one-time consultation. (Theme 3) |
| Affective Attitude | - Referring clinicians cited an overall positive response to the service. (Theme 1) - Genetic counseling offerings were helpful, and PCCs felt able to connect patients with the care they needed. (Theme 2) - Scheduling patients was timely and prompt. Many cited it was faster for their patients to be seen than in other specialty clinics. (Themes 1 and 2) - Virtual nature of the service was seen as lower burden (for patients and PCCs) and was accessible to patients. (Themes 1 and 2) - Referring PCCs perceived that counseling delivered was thorough. (Theme 1) - Service didn’t ask referring PCCs to do things outside of their perceived scope of practice, such as provide genetic counseling to the patients or disclose genetic test results. (Theme 2) - Communication from the PGCS team was good, including the quality of clinical notes and comments sent to the referring clinician summarizing the visit and any next steps. (Theme 3) |
| Perceived Effectiveness | - All referring providers endorsed that the service was effective for them. Many commented that it was effective for them because the service was effective for their patients. (Themes 1 and 2) |
| Burden of PGCS on Referring PCCs | - PCCs stated low workload burden on the referring clinician (discuss with patient and place electronic referral). (Theme 2) - Little follow-up was needed and the follow-up processes were streamlined (Theme 2) - Referring PCCs liked that the genetic counselor disclosed the test results to the patient. (Theme 2) - Not asked to do work perceived as outside of their scope of practice. (Themes 1 and 2) |
| Ethicality of the PGCS | - Referring clinicians did not feel that there was any potential harm in referring to this service compared with an alternative. (Theme 1) - Some had concerns about the risks of genetic testing, especially regarding psychological welfare and privacy, but they did not have concerns about how the PGCS was handling these issues. (Theme 1) |
| Self-efficacy | - Endorsed a high likelihood of continuing to refer to this service, although some asked for a more streamlined and clear referral process. (Themes 1 and 2) |
| Change in Perceptions of Genetic Counseling and Genetic Testing | - A few providers cited that their knowledge and comfort about the genetic counseling process had increased as a result of referring their patients to the service; however, most did not indicate any change. (Theme 2) - A single referring clinician expressed comfort counseling patients on the risk of Alzheimer’s disease amongst carriers of the *APOE4* risk allele, which was one of the main DTC test results patients were referred to the PGCS to discuss. (Theme 2) - All but 1 provider denied being able to take on genetic counseling in their practice because of the amount of time and knowledge (including evolving knowledge) needed. (Theme 2) - Few referring PCCs said that they could imagine taking on ordering genetic testing in the future, and if they did, they would require clarity about what testing they should order and additional education and training. (Themes 1 and 2) |
| Changes in Perceptions of Offering High-Risk Breast Cancer Screening | - Although most patients were referred for a family history of breast cancer, few providers felt comfortable taking on counseling about incorporating breast MRI as part of a screening strategy for high-risk individuals. (Theme 2) - No clinicians felt comfortable prescribing breast cancer risk-reducing medication such as tamoxifen without additional training. (Theme 2) |
| Existing alternatives to the PGCS | These were viewed as similar in terms of quality of genetic counseling, but slower to get into. These alternatives included: (Theme 1)   - Traditional subspecialty clinics including cancer genetics, high risk breast clinics, neurology (for AD risk) and genetics clinics. - Ordering genetic testing themselves as primary care clinician. - Requesting an electronic consultation. |
| Provider Future Needs | Referring PCCs cited the following future needs:   - Centralized referral hub where referrals would be sorted and sent to the best specialty for that question, as opposed to the PCC trying to determine the correct genetics referral. - More neurogenetics resources given increasing questions about genetic risk for Alzheimer’s disease. - Teaching about insurance and financial implications of genetic testing. - More preconception and reproductive genetic risk resources. - Support for patients with risk of hemochromatosis. - Direct-to-consumer genetic testing resources. |
| Suggested Areas for Improvement and Growth of the PGCS | Focus on equity (Theme 2)   - Consider providing group education in different languages that is culturally sensitive. - Make sure all patients can get in. - Proactively identify patients who should be seen by a genetic counselor.   Expand menu of genetic counseling and testing offerings to other disease risks. (Theme 2)   - Expand cancer susceptibility to include genetic testing for risk of other cancers, especially GI cancers. - Proactively identify patients who should be seen by a genetic counselor. - Improve communication and referral processes further. (Theme 3) - Create and disseminate fact sheet or other information about what patients should expect from a visit. - Add screening/surveillance recommendations generated because of the visit in the electronic health record’s health maintenance reminders field. - Make the location of the genetic test results in the electronic health record clearer. - Decrease re-routing of referrals. - Make clearer who is responsible for signing test orders. |

# PGCS Clinical Tracker Data Collection Fields

| **Variable /**  **Field Name** | **Field Type** | **Field Label** | **Choices, Calculations, OR Slider Labels** | **Identifier?** | **Branching Logic (Show field only if...)** | **Required Field?** |
| --- | --- | --- | --- | --- | --- | --- |
| record_id | text | Record ID |  |  |  |  |
| first_name | text | First Name |  | y |  | y |
| last_name | text | Last Name |  | y |  | y |
| mrn | text | MRN |  | y |  | y |
| birth_date | text | Date of birth |  | y |  | y |
| age_at_visit | calc | Age of patient at visit | rounddown((datediff([birth_date],[dov],"y","mdy")),0) |  |  |  |
| workqueue | radio | Workqueue source | 1, PGCS \| 2, CCRA \| 3, Other |  |  | y |
| cancerschedule | text | What date were they originally scheduled? |  |  | [workqueue] = '2' |  |
| sex | radio | Sex at birth | 1, Male \| 2, Female \| 3, Other |  |  | y |
| gender_identity | radio | Gender identity | 1, Male\|2, Female\|3, Non-binary\|4, Prefers to self-describe\|5, Unknown |  |  |  |
| specify_gender | text | Please specify gender identity |  |  | [gender_identity] = '4' |  |
| race | checkbox | Race | 1, American Indian or Alaskan Native \| 2, Asian \| 3, Asian Indian \| 4, Black or African American \| 5, Native Hawaiian or Other Pacific Islander \| 6, White or Caucasian \| 7, Other \| 8, Unknown |  |  | y |
| race_other | text | If other, please specify |  |  | [race(7)] = '1' |  |
| ethnicity | radio | Ethnicity | 1, Hispanic or Latinx \| 2, Non-Hispanic or Non-Latinx \| 3, Unknown |  |  | y |
| proband | yesno | Is this patient the proband? |  |  |  |  |
| relationship_to_proband | radio | Relationship to proband | 1, Mother \| 2, Father \| 3, Sibling (male) \| 4, Sibling (female) \| 5, Maternal Half-Sibling (male) \| 6, Maternal Half-Sibling (female) \| 7, Maternal Aunt \| 8, Maternal Uncle \| 9, Maternal 1st Cousin (male) \| 10, Maternal 1st Cousin (female) \| 11, Maternal Grandmother \| 12, Maternal Grandfather \| 13, Other Maternal Relative \| 14, Paternal Half-Sibling (male) \| 15, Paternal Half-Sibling (female) \| 16, Paternal Aunt \| 17, Paternal Uncle \| 18, Paternal 1st Cousin (male) \| 19, Paternal 1st Cousin (female) \| 20, Paternal Grandmother \| 21, Paternal Grandfather \| 22, Other Paternal Relative \| 23, Niece \| 24, Nephew \| 25, Son \| 26, Daughter \| 27, Grandson \| 28, Granddaughter \| 29, Partner |  | [proband] = '0' |  |
| family_code | text | <div class="rich-text-field-label"><p>Please enter the family code <br /><span style="font-weight: normal;">(original proband's record ID followed by "F" i.e. 1234F)</span></p></div> |  | y |  |  |
| deceased | yesno | Is this patient deceased? |  |  |  | y |
| age_at_death | text | Age at death |  |  | [deceased] = '1' | y |
| next_of_kin | text | <div class="rich-text-field-label"><p>Next of kin name<br /><span style="font-weight: normal;">(First Last)</span></p></div> |  | y | [deceased] = '1' |  |
| relationship_to_deceased | radio | Relationship to deceased | 1, Mother \| 2, Father \| 3, Sibling (male) \| 4, Sibling (female) \| 5, Maternal Half-Sibling (male) \| 6, Maternal Half-Sibling (female) \| 7, Maternal Aunt \| 8, Maternal Uncle \| 9, Maternal 1st Cousin (male) \| 10, Maternal 1st Cousin (female) \| 11, Maternal Grandmother \| 12, Maternal Grandfather \| 13, Other Maternal Relative \| 14, Paternal Half-Sibling (male) \| 15, Paternal Half-Sibling (female) \| 16, Paternal Aunt \| 17, Paternal Uncle \| 18, Paternal 1st Cousin (male) \| 19, Paternal 1st Cousin (female) \| 20, Paternal Grandmother \| 21, Paternal Grandfather \| 22, Other Paternal Relative \| 23, Niece \| 24, Nephew \| 25, Son \| 26, Daughter \| 27, Grandson \| 28, Granddaughter \| 29, Partner |  | [deceased] = '1' |  |
| me | text | Medical Examiner name/office |  |  | [deceased] = '1' |  |
| gc | radio | Genetic Counselor | [redacted] |  |  | y |
| md | radio | Physician | [redacted] |  |  | y |
| dov | text | Date of visit |  |  |  | y |
| type_of_visit | radio | Type of visit | 1, In-person\|2, Phone call\|3, Virtual/video visit |  |  | y |
| status | dropdown | Status | 1, Scheduled \| 2, Seen by clinic \| 3, Unable to reach, referral cancelled \| 4, Pt declined appointment |  |  |  |
| research | yesno | Is this visit part of a research study? |  |  |  |  |
| date_of_referral | text | Date of Referral |  |  |  |  |
| referring_prov | text | <div class="rich-text-field-label"><p>Referring Provider<br /><span style="font-weight: normal;">Last, First</span></p></div> |  |  |  |  |
| referringinstitution | radio | Referring Provider Location | [redacted] |  |  |  |
| indication | radio | Indication for referral | 1, Family history of breast cancer and related cancer \| 2, Preconception testing \| 3, Direct to consumer test interpretation \| 4, Other \| 5, Family history of Alzheimer's Disease or dementia \| 6, Adopted Patient Seeking Genetic Testing |  |  | y |
| indicationother | text | Please describe |  |  | [indication] = '4' |  |
| fhx | yesno | Is there a family history of disease related to the patient's referral indication? |  |  |  | y |
| relativeshx | checkbox | Please check off any family members that have a personal history related to the patient's referral indication | 1, Mother \| 2, Father \| 3, Sibling (male) \| 4, Sibling (female) \| 5, Maternal Half-Sibling (male) \| 6, Maternal Half-Sibling (female) \| 7, Maternal Aunt \| 8, Maternal Uncle \| 9, Maternal 1st Cousin (male) \| 10, Maternal 1st Cousin (female) \| 11, Maternal Grandmother \| 12, Maternal Grandfather \| 13, Other Maternal Relative \| 14, Paternal Half-Sibling (male) \| 15, Paternal Half-Sibling (female) \| 16, Paternal Aunt \| 17, Paternal Uncle \| 18, Paternal 1st Cousin (male) \| 19, Paternal 1st Cousin (female) \| 20, Paternal Grandmother \| 21, Paternal Grandfather \| 22, Other Paternal Relative \| 23, Niece \| 24, Nephew \| 25, Son \| 26, Daughter \| 27, Grandson \| 28, Granddaughter \| 29, Partner |  | [fhx] = '1' |  |
| consanguinity | radio | Consanguinity | 1, Yes \| 0, No \| 2, Unknown |  |  |  |
| gtdecision | yesno | Did the patient agree to pursue genetic testing? |  |  |  |  |
| considering_testing | truefalse | Patient is considering genetic testing |  |  |  |  |
| econsent | radio | eConsent Status | 1, eConsent sent \| 2, eConsent signed \| 3, eConsent sent but unsigned, patient received reminder |  | [gtdecision] = '1' |  |
| gt_ordered | yesno | Genetic Testing Ordered |  |  | [econsent] = '2' | y |
| date_genetic_test_ordered | text | Date Genetic Test was Ordered |  |  |  |  |
| test_status | dropdown | Test Status | 1, Sent to patient \| 2, Returned to lab |  |  |  |
| days_since_order_placed | calc | Days since test order placed | datediff("today",[date_genetic_test_ordered],"d") |  |  |  |
| remindersent | radio | Has a reminder been sent to return testing kit? | 1, Reminder Sent (Unread) \| 2, Reminder Sent (Read) |  |  |  |
| ntests | radio | How many tests were ordered? | 1, 1 \| 2, 2 |  | [gt_ordered] = '1' | y |
| test1 | radio | Test 1 Type | [redacted] |  | [gt_ordered] = '1' | y |
| test2 | radio | Test 2 Type | [redacted] |  | [ntests] = '2' or [ntests] = '3' | y |
| report_upload_epic | yesno | Report(s) uploaded to Epic |  |  |  |  |
| n_var | radio | <div class="rich-text-field-label"><p>Number of variants reported <br /><br /></p></div> | 0, 0\|1, 1\|2, 2\|3, 3\|4, 4\|5, 5 |  | [gt_ordered] = '1' | y |
| var1gene | text | Variant 1: Gene symbol |  |  | [n_var] = '1' or [n_var] = '2' or [n_var] = '3' or [n_var] = '4' or  [n_var] = '5' | y |
| var1cdna | text | Variant 1: cDNA change |  |  | [n_var] = '1' or [n_var] = '2' or [n_var] = '3' or [n_var] = '4' or  [n_var] = '5' | y |
| variant_1_protein | text | Variant 1: Protein change |  |  | [n_var] = '1' or [n_var] = '2' or [n_var] = '3' or [n_var] = '4' or  [n_var] = '5' | y |
| variant_1_interpretation | radio | Variant 1: Interpretation | 1, Path\|2, Likely Path\|3, VUS\|4, Likely Benign\|5, Benign |  | [n_var] = '1' or [n_var] = '2' or [n_var] = '3' or [n_var] = '4' or  [n_var] = '5' | y |
| var1genomcoord | text | <div class="rich-text-field-label"><p>Variant 1: Genomic coordinate OR transcript ID</p> <ul> <li><span style="font-weight: normal;">For genomic coordinate, please use format:</span><br /><span style="font-weight: normal;">"11:27901111"</span></li> <li><span style="font-weight: normal;">For transcript ID, please use format:</span><br /><span style="font-weight: normal;">"NM_000218.2"</span></li> </ul></div> |  |  | [n_var] = '1' or [n_var] = '2' or [n_var] = '3' or [n_var] = '4' or  [n_var] = '5' | y |
| var2gene | text | Variant 2: Gene symbol |  |  | [n_var] = '2' or [n_var] = '3' or [n_var] = '4' or  [n_var] = '5' | y |
| var2cdna | text | Variant 2: cDNA change |  |  | [n_var] = '2' or [n_var] = '3' or [n_var] = '4' or  [n_var] = '5' | y |
| variant_2_protein | text | Variant 2: Protein change |  |  | [n_var] = '2' or [n_var] = '3' or [n_var] = '4' or  [n_var] = '5' | y |
| variant_2_interpretation | radio | Variant 2: Interpretation | 1, Path\|2, Likely Path\|3, VUS\|4, Likely Benign\|5, Benign |  | [n_var] = '2' or [n_var] = '3' or [n_var] = '4' or  [n_var] = '5' | y |
| var2genomcoord | text | <div class="rich-text-field-label"><p>Variant 2: Genomic coordinate or transcript ID</p> <ul> <li><span style="font-weight: normal;">For genomic coordinate, please use format:</span><br /><span style="font-weight: normal;">"11:27901111"</span></li> <li><span style="font-weight: normal;">For transcript ID, please use format:</span><br /><span style="font-weight: normal;">"NM_000218.2"</span></li> </ul></div> |  |  | [n_var] = '2' or [n_var] = '3' or [n_var] = '4' or  [n_var] = '5' | y |
| var3gene | text | Variant 3: Gene symbol |  |  | [n_var] = '3' or  [n_var] = '4' or  [n_var] = '5' | y |
| var3cdna | text | Variant 3: cDNA change |  |  | [n_var] = '3' or  [n_var] = '4' or  [n_var] = '5' | y |
| variant_3_protein | text | Variant 3: Protein change |  |  | [n_var] = '3' or  [n_var] = '4' or  [n_var] = '5' | y |
| variant_3_interpretation | radio | Variant 3: Interpretation | 1, Path\|2, Likely Path\|3, VUS\|4, Likely Benign\|5, Benign |  | [n_var] = '3' or  [n_var] = '4' or  [n_var] = '5' | y |
| var3genomcoord | text | <div class="rich-text-field-label"><p>Variant 3: Genomic coordinate or transcript ID</p> <ul> <li><span style="font-weight: normal;">For genomic coordinate, please use format:</span><br /><span style="font-weight: normal;"> "11:27901111"</span></li> <li><span style="font-weight: normal;">For transcript ID, please use format:</span><br /><span style="font-weight: normal;">"NM_000218.2"</span></li> </ul></div> |  |  | [n_var] = '3' or  [n_var] = '4' or  [n_var] = '5' | y |
| var4gene | text | Variant 4: Gene symbol |  |  | [n_var] = '4' or  [n_var] = '5' | y |
| var4cdna | text | Variant 4: cDNA change |  |  | [n_var] = '4' or  [n_var] = '5' | y |
| variant_4_protein | text | Variant 4: Protein change |  |  | [n_var] = '4' or  [n_var] = '5' | y |
| variant_4_interpretation | radio | Variant 4: Interpretation | 1, Path\|2, Likely Path\|3, VUS\|4, Likely Benign\|5, Benign |  | [n_var] = '4' or  [n_var] = '5' | y |
| var4genomcoord | text | <div class="rich-text-field-label"><p>Variant 4: Genomic coordinate or transcript ID</p> <ul> <li><span style="font-weight: normal;">For genomic coordinate, please use format:</span><br /><span style="font-weight: normal;">"11:27901111"</span></li> <li><span style="font-weight: normal;">For transcript ID, please use format:</span><br /><span style="font-weight: normal;">"NM_000218.2"</span></li> </ul></div> |  |  | [n_var] = '4' or  [n_var] = '5' | y |
| var5gene | text | Variant 5: Gene symbol |  |  | [n_var] = '5' | y |
| var5cdna | text | Variant 5: cDNA change |  |  | [n_var] = '5' | y |
| variant_5_protein | text | Variant 5: Protein change |  |  | [n_var] = '5' | y |
| variant_5_interpretation | radio | Variant 5: Interpretation | 1, Path\|2, Likely Path\|3, VUS\|4, Likely Benign\|5, Benign |  | [n_var] = '5' | y |
| var5genomcoord | text | <div class="rich-text-field-label"><p>Variant 5: Genomic coordinate or transcript ID</p> <ul> <li><span style="font-weight: normal;">For genomic coordinate, please use format:</span><br /><span style="font-weight: normal;">"11:27901111"</span></li> <li><span style="font-weight: normal;">For transcript ID, please use format:</span><br /><span style="font-weight: normal;">"NM_000218.2"</span></li> </ul></div> |  |  | [n_var] = '5' | y |
| report_date | text | Test 1: Report date |  |  | [ntests] = '1' or [ntests] = '2' or [ntests] = '3' or [ntests] = '4' | y |
| test1broad | radio | Test 1: Overall Result Interpretation | 1, Positive\|0, Negative\|2, Inconclusive\|3, Other |  | [gt_ordered] = '1' | y |
| test1var | checkbox | Variants reported with test 1 | 1, Variant 1\|2, Variant 2\|3, Variant 3\|4, Variant 4\|5, Variant 5 |  | [ntests] = '2' or [ntests] = '3' |  |
| test_2_report_date | text | Test 2: Report date |  |  | [ntests] = '2' or [ntests] = '3' or [ntests] = '4' | y |
| test2_broad | radio | Test 2: Overall Result Interpretation | 1, Positive\|0, Negative\|2, Inconclusive\|3, Other |  | [ntests] = '2' or [ntests] = '3' or [ntests] = '4' | y |
| test2var | checkbox | Variants reported with test 2 | 1, Variant 1\|2, Variant 2\|3, Variant 3\|4, Variant 4\|5, Variant 5 |  | [ntests] = '2' |  |
| var_reclass | yesno | Were any variants reclassified? |  |  | [n_var] = '1' or [n_var] = '2' or [n_var] = '3' or [n_var] = '4' or [n_var] = '5' |  |
| please_denote_any_changes | notes | <div class="rich-text-field-label"><p>Please denote variant reclassification in the following format: <br /><span style="font-weight: normal;">Reinterpretation date, variant number (1-5), original interpretation->updated interpretation.</span></p> <p><span style="font-weight: 400;">ex:</span></p> <p><span style="font-weight: 400;">03-25-2020, 1, VUS->P.</span></p> <p><span style="font-weight: 400;">03-27-2020, 3, VUS->LB.</span></p> <p> </p> <p>Please update the original variant entry to reflect the new classification.</p></div> |  |  | [var_reclass] = '1' |  |
| tccalculated | yesno | TC Score Calculated? |  |  | [indication] = '1' |  |
| tcscore | text | TC Score |  |  | [indication]='1' |  |
| date_result_disclosed | text | Date results disclosed |  |  | [gt_ordered] = '1' | y |
| nccn_criteria | yesno | Meet NCCN Criteria by fam hx |  |  |  |  |
| result_discloser | radio | Who disclosed the results | [redacted] |  | [gt_ordered] = '1' |  |
| how_result_was_disclosed | radio | How results were disclosed | 1, Phone call \| 2, In-person \| 3, Virtual/Video visit \| 4, Letter, Patient Gateway, or through Testing Company Portal (no phone or video contact) |  | [gt_ordered] = '1' |  |
| gc_notes | notes | Genetic counseling notes: |  |  |  |  |
| referralnecessary | radio | Referral to another clinic necessary? | 1, Avon \| 2, CCRA \| 3, Medical Genetics \| 4, OB Genetics \| 5, Other \| 6, No referral necessary |  |  |  |
| referralscheduled | yesno | Has patient been scheduled for this referral? |  |  | [referralnecessary] = '1' or [referralnecessary] = '2' or [referralnecessary] = '3' |  |
| referralplaced | yesno | Is there documentation in the chart that either the referral was co-signed or the provider actively decided not to send a referral? |  |  | [referralscheduled] = '0' |  |
| billing_type | radio | Billing Type | 1, Insurance\|2, Patient-Pay\|3, Institutional\|4, Sponsored Testing Program |  |  | y |
| primary_cvg | text | Patient Insurance plan |  |  | [billing_type] = '1' |  |
| prior_auth_needed | yesno | Prior Authorization needed? |  |  | [billing_type] = '1' |  |

# Patient-facing survey

**The MGH Preventive Genetic Counseling Service:**

**Patient Survey (to be Programmed in REDCap for Administration)**

**Introduction:**

We invite you to complete this survey about your experiences with the MGH Preventive Genetic Counseling Service. This survey is being conducted as part of a research study to understand the impact of the MGH Preventive Genetic Counseling Service pilot. A study invitation and fact sheet was sent to you along with the link to this survey.

**By completing this survey, you have consented for your responses to be included in the research study.**

We anticipate that this survey will take less than 10 minutes to complete. You will have the option to receive an Amazon Gift Card in thanks for completing the survey.

If you have any questions or comments about the study, contact the Principal Investigator, Leland Hull, MD, at [lhull1@mgh.harvard.edu](mailto:lhull1@mgh.harvard.edu). The study team’s contact information is also available in the Study Fact Sheet you received with the survey link.

Please indicate whether you would like to be included in this study. If you would not like to be included in this study and would like to opt out of future reminders about this survey, please select No. This will ensure that no further survey reminders will be sent.

- Yes, I do want to be included in this study
- No, I do not want to be included in this study

**Survey Questions:**

|  | **Question Stem** | **Response Options** |
| --- | --- | --- |
| I. | [Section I. Patient Motivation.] We would like to learn a little more about your motivation(s) for visiting with the genetic counselor. | |
| I.1. | What motivated you to meet with the genetic counselor? Check all that apply.^1^ | - My clinician recommended it. - To learn about my personal risk of disease. - To learn about the risk of disease in my family. - To satisfy my curiosity. - To better understand the results of testing I did somewhere else. - Other - Prefer not to answer |
| I.2. | [If “Other”]: Please describe. | Free response. |
| II.1. | [Section II. Patient Satisfaction]. Next, we would like to learn a bit more about whether you were satisfied with the services provided by the clinic. | |
| II.2. | Was the visit as soon as you needed?^2^ | - Yes, definitely - Yes, somewhat - No - Prefer not to answer |
| II.3. | Did your visit start on time?^2^ | - Yes, definitely - Yes, somewhat - No - Prefer not to answer |
| II.4. | Did the genetic counselor explain things in a way that was easy to understand? ^2^ | - Yes, definitely - Yes, somewhat - No - Prefer not to answer |
| II.5. | Did the genetic counselor listen carefully to you?^2^ | - Yes, definitely - Yes, somewhat - No - Prefer not to answer |
| II.6. | Did the genetic counselor show respect for what you had to say?^2^ | - Yes, definitely - Yes, somewhat - No - Prefer not to answer |
| II.7. | Did the genetic counselor spend enough time with you?^2^ | - Yes, definitely - Yes, somewhat - No - Prefer not to answer |
| II.8. | Prior to your visit, a staff member called to schedule you. Was the staff as helpful as you thought they should be? | - Yes, definitely - Yes, somewhat - No - Prefer not to answer |
| II.9. | Did the staff treat you with courtesy and respect? | - Yes, definitely - Yes, somewhat - No - Prefer not to answer |
| II.10. | On a scale of 0 to 10, how likely are you to recommend the genetic counseling service to your friends or family?^3^ | - 0-10 score |
| III. | [Section III. Outcomes of genetic testing.] | |
| III.1. | [If the patient declined genetic testing at their visit] Why did you decide against getting genetic testing? Select all that apply.^4^ | - I was concerned about the cost of testing. - I was concerned about privacy. - I was fearful of insurance discrimination. - I was concerned about emotional distress due to testing. - I never received my test kit. - The process of completing the test was too difficult. - I did not feel it was necessary. - Other - Prefer not to answer |
| III.2. | [If “Other”] Please describe. | - Free response. |
| III.3. | [If the patient indicated they want to pursue genetic testing at their visit, but did not complete genetic testing within 3 months of their visit]: At your visit, you indicated that you would like to proceed with genetic testing. Why haven't you completed genetic testing yet? | - Free response |
|  | [If the patient decided against or did not complete genetic testing, skip to Section V.] | |
| III.4. | How did you learn about your genetic test results? [Select all that apply]. | - Telephone call from the clinical team - Written message from the clinical team, such as a letter or an electronic message - The test company’s website - Other - Prefer not to answer |
| III.5. | If “Other,” please describe. | Free response. |
| III.6. | Were you satisfied with how your results were communicated to you? | - Yes, definitely - Yes, somewhat - No - Prefer not to answer |
| III.7. | [If “Yes, somewhat” or “No”] Please share how we could improve the process of communicating your results with you. | Free Response. |
| IV. | [Section IV. Perceived Utility of Genetic Testing]. Next, we would like to understand if you believe the genetic testing you completed was useful. Please indicate below whether you agree or disagree with the following statements: | |
| IV.1. | I believe that my genetic test results will help me to plan better for the future.^5^ | - Strongly Agree - Agree - Neutral - Disagree - Strongly Disagree - Prefer not to answer |
| IV.2. | My genetic testing results have caused me to feel relieved. | - Strongly Agree - Agree - Neutral - Disagree - Strongly Disagree - Prefer not to answer |
| IV.3. | My genetic test results gave me the information I wanted to know. | - Strongly Agree - Agree - Neutral - Disagree - Strongly Disagree - Prefer not to answer |
| IV.4. | My genetic test results will be useful to my family members. | - Strongly Agree - Agree - Neutral - Disagree - Strongly Disagree - Prefer not to answer |
| IV.5. | My genetic test results identified a cause for a health condition I have. | - Strongly Agree - Agree - Neutral - Disagree - Strongly Disagree - Prefer not to answer |
| IV.6. | My genetic test results will influence my reproductive decisions. | - Strongly Agree - Agree - Neutral - Disagree - Strongly Disagree - Prefer not to answer |
| V. | [Section V. Patient Characteristics]. Next, we would like to learn a little bit more about how you. | |
| V.1. | In general, how would you rate your overall health now?^2,6^ | - Excellent - Very good - Good - Fair - Poor |
| V.2. | What is the highest education level you have completed? Please select one. | - Less than a high school diploma - High school diploma or GED - Some college - 2-year college (Associate's Degree) - 4-year college (Bachelor's Degree) - Master's degree - Professional Degree - Prefer not to answer |
| V.3. | [Final question]. Thank you for answering our questions. If you have any further comments that you would like to leave about your experience with the MGH Preventive Genetic Counseling Service, please take a moment to write those here. | Free response (paragraph) box. |
| End. | Thank you for taking the time to complete this survey. There are no further surveys involved in this research study.  Please enter the email address where you would like us to send you electronic $10 Amazon Gift Card, in thanks for responding to our survey. [Free response] | |

**Source of Survey Questions**

1. Sanderson, S. C. *et al.* Motivations, concerns and preferences of personal genome sequencing research participants: Baseline findings from the HealthSeq project. *Eur. J. Hum. Genet.* **24**, 14–20 (2016).

2. CAHPS® clinician & group survey 4.0 - adult. https://yourcahps.rand.org/surveys/19.

3. Reichheld, F. F. The one number you need to grow. *Harv. Bus. Rev.* **81**, 46–54, 124 (2003).

4. Robinson, J. O. *et al.* Participants and study decliners’ perspectives about the risks of participating in a clinical trial of whole genome sequencing. *J. Empir. Res. Hum. Res. Ethics* **11**, 21–30 (2016).

5. Smith, H. S. *et al.* Patient and clinician perceptions of precision cardiology care: Findings from the HeartCare Study. *Circ. Genom. Precis. Med.* **15**, e003605 (2022).

6. Ware, J. E., Jr & Gandek, B. Overview of the SF-36 Health Survey and the International Quality of Life Assessment (IQOLA) Project. *J. Clin. Epidemiol.* **51**, 903–912 (1998).

7. Haun, J., Luther, S., Dodd, V. & Donaldson, P. Measurement variation across health literacy assessments: implications for assessment selection in research and practice. *J. Health Commun.* **17 Suppl 3**, 141–159 (2012).

# Qualitative Interview Guide

**The MGH Preventive Genetic Counseling Service Pilot Evaluation:**

**Interview Guide Tailored to Clinicians Referring to the Genetic Counseling Service**

**Introduction and Fact Sheet Review:**

[This section is not audio-recorded. Please read verbatim to the participant]:

***Thank you for agreeing to meet with us and for your time today. Before we get started, I want to confirm that you received and were able to review the Study Fact sheet attached to the email about this focus group [or interview]. Does anyone have any questions about the study or the contents of the Fact Sheet?***

[Wait for response. If they have not reviewed the Study Fact sheet, ask them to take the time to review it. The interviewer can also share their screen and display the study fact sheet.]

***Do you have any questions before we get started?***

[Wait for response.]

***Okay, let’s get started. We will be audio and video recording from this point forward as we describe the study and review verbal consent language. I will now turn on the recorder.***

**Introduction and Recording Consent:**

[Please start by stating the participant ID, the interviewer’s name(s), and the date for the record.]

[Please read verbatim to the participant]:

***Thank you again. The purpose of today’s interview is to better understand your experiences referring to the MGH Preventive Genetic Counseling Service. Your participation in this interview is voluntary. The interview will be audio and video recorded and the audio will be transcribed. The risks and benefits of participating and the study procedures were outlined in the Study Fact Sheet you reviewed. You may refuse to answer any questions and you can discontinue your participation at any time.***

***Do you consent to participate in this interview?***

*[Wait for response.]*

***Thanks so much. One of the reasons you were asked to interview is because you have referred multiple patients to this new service. Before we ask you any questions, we want to remind you which of your patients were referred to our service. You were sent this information in an email, and we will share it with you again now.*** *[Moderator shares screen with recruitment email that lists the patients that provider had sent to the service. Then the moderator moves on to the questions below.]*

*[For the Moderator: For each question, there are core questions with optional italicized prompts. Please use the core questions and prompts as you see fit based on the flow of the focus group conversation as you see fit.* ***The bolded questions are more important, if time is running short.****]*

| **Question and Prompts** | **Construct Addressed** |
| --- | --- |
| - **Tell me/us a little bit about the patients/patient population seen in your practice.**    - *Male, female predominant?*   - *Languages spoken?*   - *Chronically ill, more preventive care, mix?*   - *Insurance coverage? MassHealth, private insurance, Medicare?*   - *Other features?* | Patient Needs and Population |
| - **Tell me about a patient/the patients you sent to the genetic counseling service. Why did you send the patient(s)? *What problems were you hoping the service could solve?*** - **Can you tell me a bit about your experience using the service?** [This could also be the Affective Attitude domain below.]   - - *Who started the discussion about genetic testing, you or the patient?*     - *Walk me through the process and what happened to your patient. Was the patient’s issue resolved? If no, why not? What do you like? What don’t you like? Are there any changes that you would suggest?* | Experience with the service |
| - [Intervention coherence] **Do you feel like you understand how this service works? Is the process your patient went through transparent to you? Tell me why or why not.** - [Affective attitude] How do you feel about the genetic counseling service after your experiences? - [Perceived Effectiveness] **Do you believe that the genetic counseling service has been effective?**   - *For you? For your patients? Why or why not? Do you believe that the genetic counseling service has been effective for the care of your patient? Why or why not? Did using this service generate more work for you?* - [Burden] **How much effort did the genetic counseling service referral require to have your patient seen?** *Was it too much? About right? Did the patient contact you after the referral (or testing)? If so, how much effort did receiving the genetic testing service results require? Too much? About right?* - [Opportunity costs] **Did you feel that by using this service you or your patient were giving up any benefits they would have gotten if they sought care for their concern(s) elsewhere?** *Please describe if so*. - [Self-efficacy] **How confident are you that you could continue to refer to this service?** *Are there any modifications needed so that you would feel more confident.* - [Ethicality] Did you have any ethical concerns about sending your patients to this service? *For example, compared with referring to another specialty service? Did you have any concerns about the care or service provided?* | Acceptability of the service (constructs including affective attitude, burden, ethicality, intervention coherence, opportunity costs, perceived effectiveness, self-efficacy) |
| - **If this genetic counseling service did not exist, how would you have managed the patients you referred to this service instead?** What would you have done about the concerns for which they were referred? | Alternatives |
| - Please describe if and how your perceptions of genetic counseling and genetic testing changed after using this service.   - *Do you feel more/less comfortable with genetic counseling and the genetic testing process after using this service? Please describe.* - **Could you envision yourself counseling about or ordering genetic testing for your patients seen by this service in the future?** *Why or why not?* - For providers referring patients for breast cancer risk only: **Currently, we are offering to send patients who have negative genetic testing, but an elevated lifetime risk of breast cancer (20% or greater) to the Avon Breast Clinic to discuss enhanced screening with Breast MRI and/or, depending on age and risk, use of risk reducing medications like tamoxifen. Could you see yourself counseling your patients about breast MRI and/or risk reducing medications in the future?**   - *If yes, what resources would make it easier for you to incorporate these services into your clinical practice?*   *If no, why not? What are the main barriers?* | Perceptions of Genetic Counseling and Genetic Testing |
| - **Are there other genetic counseling services that you think your patients would benefit from instead of in addition to the services offered?**   - *What problems/gaps in care would these services address?* - Are you able to see an increase in the number of patients who could benefit from genetic counseling? (quantify if possible >10/month?) - Are there other genetics services that you usually use? What do you like/not like about these services? - **Are there other ways this service could be delivered that you would prefer?** *For example, as shared medical appointments, or by referring to a champion doctor or APP in your own practice?* | Provider Needs |

**This concludes our prepared questions for today. What would you like to add that we didn’t ask about?**

[Wait for response.]

**Thank you again for participating. To thank you for your participation, we will be sending you an emailed link for a $50 Amazon Gift Card.**

**We look forward to using this feedback to improve our services and clinical offerings. Our team’s contact information is on the Study Fact Sheet if you want to be in touch. Thank you again!**

# COREQ Checklist

| **Topic** | **Item No.** | **Guide Questions/Description** | **Section Where Reported** |  |
| --- | --- | --- | --- | --- |
| **Domain 1: Research team and reflexivity** | | | |  |
| *Personal characteristics* | | | |  |
| Interviewer/facilitator | 1 | Which author/s conducted the interview or focus group? | Methods |  |
| Credentials | 2 | What were the researcher’s credentials? E.g. PhD, MD | Methods and Author Info |  |
| Occupation | 3 | What was their occupation at the time of the study? | Methods |  |
| Gender | 4 | Was the researcher male or female? | Not Reported |  |
| Experience and training | 5 | What experience or training did the researcher have? | Methods |  |
| *Relationship with participants* | | | |  |
| Relationship established | 6 | Was a relationship established prior to study commencement? | No |  |
| Participant knowledge of  the interviewer | 7 | What did the participants know about the researcher? e.g. personal goals, reasons for doing the research | Interview Guide in Supplement |  |
|  |  |  |  |  |
|  |  |  |  |  |
| Interviewer characteristics | 8 | What characteristics were reported about the interviewer/facilitator? e.g. bias, assumptions, reasons and interests in the research topic | Interview Guide in Supplement |  |
|  |  |  |  |  |
|  |  |  |  |  |
| **Domain 2: Study design** | | | | |
| *Theoretical Framework* | | | |  |
| Methodological orientation and Theory | 9 | What methodological orientation was stated to underpin the study? e.g. grounded theory, discourse analysis, ethnography, phenomenology,  content analysis | Methods |  |
|  |  |  |  |  |
|  |  |  |  |  |
| *Participant selection* | | | | |
| Sampling | 10 | How were participants selected? e.g. purposive, convenience, consecutive, snowball | Methods |  |
|  |  |  |  |  |
|  |  |  |  |  |
| Method of approach | 11 | How were participants approached? e.g. face-to-face, telephone, mail, Email | Methods |  |
|  |  |  |  |  |
|  |  |  |  |  |
| Sample size | 12 | How many participants were in the study? | Results |  |
| Non-participation | 13 | How many people refused to participate or dropped out? Reasons? | Results |  |
| *Setting* | | | |  |
| Setting of data collection | 14 | Where was the data collected? e.g. home, clinic, workplace | Methods |  |
| Presence of non-  participants | 15 | Was anyone else present besides the participants and researchers? | No |  |
|  |  |  |  |  |
|  |  |  |  |  |
| Description of sample | 16 | What are the important characteristics of the sample? e.g. demographic data, date | Methods |  |
|  |  |  |  |  |
|  |  |  |  |  |
| *Data collection* | | | |  |
| Interview guide | 17 | Were questions, prompts, guides provided by the authors? Was it pilot tested? | Methods |  |
|  |  |  |  |  |
| Repeat interviews | 18 | Were repeat interviews carried out? If yes, how many? | Methods – None |  |
| Audio/visual recording | 19 | Did the research use audio or visual recording to collect the data? | Methods |  |
| Field notes | 20 | Were ﬁeld notes made during and/or after the interview or focus group? | No |  |
| Duration | 21 | What was the duration of the interviews or focus group? | Methods |  |
| Data saturation | 22 | Was data saturation discussed? | Methods |  |
| Transcripts returned | 23 | Were transcripts returned to participants for comment and/or correction? | No |  |
| **Domain 3: analysis and ﬁndings** | | | |  |
| *Data analysis* | | | |  |
| Number of data coders | 24 | How many data coders coded the data? | Methods |  |
| Description of the coding  tree | 25 | Did authors provide a description of the coding tree? | Methods |  |
| Derivation of themes | 26 | Were themes identiﬁed in advance or derived from the data? | Methods |  |
| Software | 27 | What software, if applicable, was used to manage the data? | Methods |  |
| Participant checking | 28 | Did participants provide feedback on the ﬁndings? | No |  |
| *Reporting* | | | |  |
| Quotations presented | 29 | Were participant quotations presented to illustrate the themes/ﬁndings? Was each quotation identiﬁed? e.g. participant number | Results |  |
| Data and ﬁndings consistent | 30 | Was there consistency between the data presented and the ﬁndings? | Results |  |
| Clarity of major themes | 31 | Were major themes clearly presented in the ﬁndings? | Results |  |
| Clarity of minor themes | 32 | Is there a description of diverse cases or a discussion of minor themes? | No |  |
